# Functional effects of human milk oligosaccharides (HMOs)

**DOI:** 10.1080/19490976.2023.2186115

**Published:** 2023-03-17

**Authors:** Meltem Dinleyici, Jana Barbieur, Ener Cagri Dinleyici, Yvan Vandenplas

**Affiliations:** a Department of Social Pediatrics, Eskisehir Osmangazi University Faculty of Medicine, Eskisehir, Turkey; b UZ Brussel, KidZ Health Castle, Vrije Unversiteit Brussel, Brussels, Belgium; c Department of Pediatrics, Eskisehir Osmangazi University Faculty of Medicine, Eskisehir, Turkey

**Keywords:** Human milk oligosaccharide, HMO, human milk, breastfeeding, microbiota

## Abstract

Human milk oligosaccharides (HMOs) are the third most important solid component in human milk and act in tandem with other bioactive components. Individual HMO levels and distribution vary greatly between mothers by multiple variables, such as secretor status, race, geographic region, environmental conditions, season, maternal diet, and weight, gestational age and mode of delivery. HMOs improve the gastrointestinal barrier and also promote a bifidobacterium-rich gut microbiome, which protects against infection, strengthens the epithelial barrier, and creates immunomodulatory metabolites. HMOs fulfil a variety of physiologic functions including potential support to the immune system, brain development, and cognitive function. Supplementing infant formula with HMOs is safe and promotes a healthy development of the infant revealing benefits for microbiota composition and infection prevention. Because of limited data comparing the effect of non-human oligosaccharides to HMOs, it is not known if HMOs offer an additional clinical benefit over non-human oligosaccharides. Better knowledge of the factors influencing HMO composition and their functions will help to understand their short- and long-term benefits.

## GRAPHICAL ABSTRACT

**Figure:**
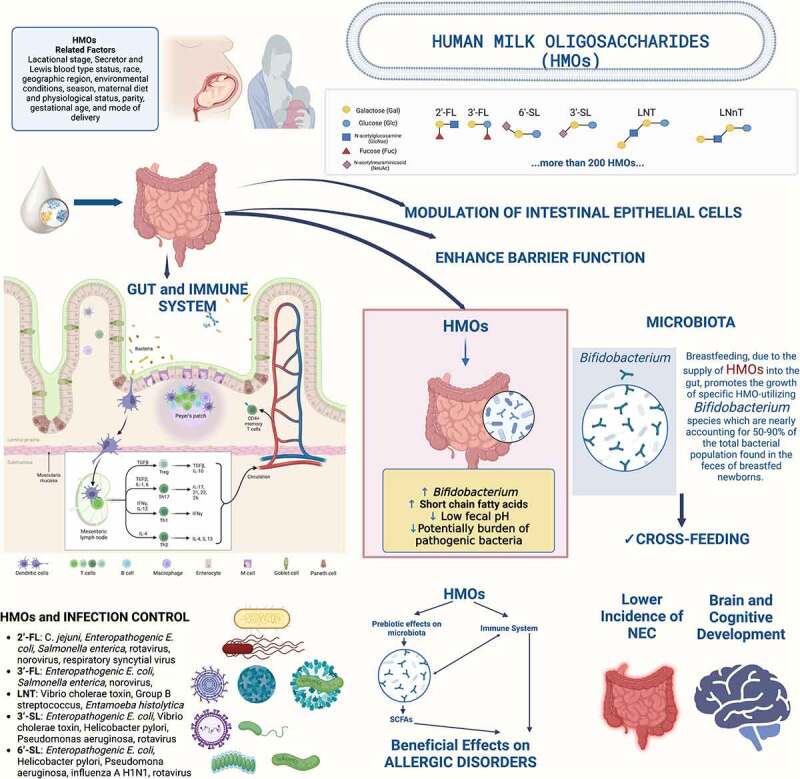

## Introduction

The World Health Organization (WHO) and pediatric societies recommend breastfeeding within the first hour of life and to breastfeed exclusively for the first six months, continuing for up to two years^1–3^. Human milk is the only recommended source of nutrition for newborns because of its unique composition and the fact that it is naturally occurring and ideally suited to support crucial developmental processes in infancy. In addition, to providing essential nutrients, human milk also contains a plethora of bioactive components that promote healthy growth and development and help to preserve a healthy microbiota and the infant’s immune system.^4–6^. There are numerous health benefits associated with breastfeeding and human milk, both for mothers (lower risks of breast and ovarian cancer, hypertension, and type 2 diabetes) and their newborns (short- and long term). Short-term benefits include fewer cases of diarrhea, pneumonia, otitis media, atopic dermatitis, and sudden infant death syndrome; long-term benefits include fewer cases of type 2 diabetes, leukemia, autistic spectrum disorders, and obesity; and beneficial effects on IQ and social behavior^5,7–13^.

The difference between non-breastfed and breastfed infants in morbidity and mortality was hypothesized to be related to the composition of human milk. The relationship between breastfeeding and infant’s health is based on its nutritional and bioactive components including human milk oligosaccharides (HMOs)^4,14,15^. In the early 1900s, Moro and Tissier independently found a predominance of bifidobacteria in the stools of breastfed compared to non-breastfed infants^16^. It was discovered that the oligosaccharides present in human milk did stimulate the growth of bifidobacteria, and in the 1950s the first clear description of the structure of the most abundant HMOs were unraveled^17–19^.

HMOs provide a variety of physiologic functions, including the establishment of a balanced infant’s gut microbiota, the strengthening of the gastrointestinal barrier, prevention of infections, and potential support to the immune system, brain, and cognitive development^4–6,14,15^. This review aims to summarize up-to-date information about the functional effects of HMOs, such as supporting the development of a healthy gastro-intestinal microbiome, inhibiting the adhesion of pathogens, promoting the development of a balanced the immune system, and their contribution to brain development and cognitive function.

## Method

We searched for relevant studies published in the English language in PubMed, EmBase, Scopus between 2000 and August 2022. We used search terms: “human milk oligosaccharide” AND “breast feeding”, OR “breastfed”, OR “human milk”, OR “formula”, OR “infant formula” and OR “nutrition”. We researched the relevant literature and summarized the most up-to-date information about the functional effects of HMOs, as well as, evaluated preclinical, observational, and randomized controlled clinical trials with HMO-containing infant formulas.

### Human Milk Oligosaccharides (HMOs): composition and related factors

Human milk contains numerous structurally different oligosaccharides, indigestible carbohydrates for humans. Human milk contains much more oligosaccharides than the milk of any animal. Human milk oligosaccharides (HMOs) are the third most important solid component in human milk after lactose and lipids, while having a minimal nutritional value for the infant^4–6,20,21^. Over 200 structurally different HMOs have currently been identified^20,22^. HMOs withstand both heat and cold, and remain therefore unaffected by pasteurization and freeze-drying^23^. HMOs are resistant to pancreatic and brush border enzymes, as well as to the low stomach pH. The majority of HMOs are either metabolized by the infant’s gut microbiota or excreted intact. Approximately, 1 to 2% of the ingested HMOs are absorbed, get into the systemic circulation, and are eliminated via urine^14^.

HMOs are multifunctional, unconjugated, and non-digestible glycans. HMOs are build out of five monosaccharide components: galactose, glucose, fucose, N-acetylglucosamine, and the sialic acid derivative N-acetyl-neuraminic acid^14,15,24^. Abbreviations of common HMOs were shown in Table 1.

**Table 1.** Abbreviation of HMOs.

2′-FL	2′fucosyllactose 2
3′-SL	3′sialyllactose
6′-SL	6′sialyllactose
DFL	2,3-di-O-fucosyllactose
DFLac	difucosyllactose
DFLNH	difucosyllacto-N-hexaose
DFLNT	difucosyllacto-*N*-tetrose
DSLNH	disialyllacto-*N*-hexaose
DSLNT	Disialyllacto-N-tetraose
FDSLNH	fucodisialyllacto-N-hexaose
FLNH	fucosyllacto-N-hexaose
LNDFH-I	lacto-N-difucohexaose:
LNFP I	lacto-N-fucopentaose I
LNFP II	lacto-N-fucopentaose II
LNFP-III	lacto-N-fucopentaose III
LNH	lacto-N-hexaose
LNnT	Lacto-N-neotetraose
LNT	Lacto-N-tetraose
LSTb	sialyl-lacto-N-tetraose b
LSTc	sialyl-lacto-N-tetraose c

Three major HMO categories are present in human milk of secretor mothers^6,14,15,25,26^

1. Neutral fucosylated HMOs (35–50%; e.g., 2′-FL and DFL)
2. Acidic sialylated HMOs (12–14%) e.g., 3′-SL and 6′-SL
3. Neutral non-fucosylated HMOs (42–55%, e.g., LNnT, LNT).

The levels and distribution of HMOs vary widely from woman to woman but also for a single woman according to the duration of lactation and many other variables (such as regional, seasonal etc.)^5,27,28^. Conze *et* al^27^ performed a weighted analysis of 2′-FL, 3′- FL, LNT, 3′-SL, and 6′-SL concentrations in human milk from previously published reports and reported the following median (± standard deviation) levels: for 2’-FL: 2.56 ± 0.054 (IQR 1.14–3.89 g/L), for 3’-FL a median of 0.32 ± 0.045 (IQR 0.057–1.1 g/L), for LNT 0.82 ± 0.0057 (IQR 0.35–1.5 g/L), for 3’-SL 0.23 ± 0.0018 (IQR 0.10–0.42 g/L) and for 6’-SL 0.33 ± 0.003 (IQR 0.09–0.54 g/L)^27^.

HMOs range in concentration from 20 to 25 g/L (average 9–22 g/L) in colostrum to 10–15 g/L (average 8–19 g/L) in mature milk, and 4–6 g/L after 6 months^15,22,29–34^. About 10 grams of HMOs are consumed daily by a term infant ingesting 800 milliliters of human milk^26^.

Individual HMO concentrations vary by secretor status and Lewis blood-type status, race, geographic region, ethnicity, environmental conditions, season, maternal diet, physiological status, parity, gestational age, and mode of delivery^4,5,14,15,28,32,33,35–41^. In secretor women (account for 70–80% of all women), 2′-FL is the most prevalent HMO, and persists at around 1 g/L after one year^35,36^. Most HMO concentrations decrease over the course of lactation. However, some HMOs, including 3’-SL, 3’-FL, and DSLNT increase in concentration throughout the first months of breastfeeding and even beyond one year of lactation^4,30,33,37^. Recent research by Plows and colleagues^33^ examined HMO levels over two-years and confirmed that the majority of HMO concentrations decrease significantly over the course of lactation among Hispanic mothers in the United States, with the exception of 2’-FL, LSTb, and DSLNT, which showed no change, as well as a 10-fold increase of 3’-FL, and a 2-fold increase of 3’-SL from the first month to the 24^th^ month of lactation. Although it is not known if these variations in HMO-levels have a clinical impact, the stability or growth of certain HMOs during lactation suggests that they may have crucial biological activities^33^.

Maternal secretor and Lewis blood-type status affect HMO fucosylation. Le gene encodes Lewis blood group antigens (FUT3 gene) and generates fucosylated HMOs in mammary glands. Se is another HMOs-related gene^5,42^. Se and Le genes encode mammary gland enzymes FUT2 and FUT3 involved in fucosylated HMO production. Se and Le genes encode FUT2 and FUT3, which classify lactating mothers into four types^14^. Lactating mothers who express active FUT2 are called “secretors,” and their milk is rich in 2′-FL and LNFP I. Non-secretors are lactating mothers who do not express active FUT2. Their milk contains few or no 1–2 fucosylated HMOs, including 2′-FL^14^. Variations in FUT2 negative genotypes contribute to geographic variances in HMO profiles^43^. Secretor mothers have greater mean total HMO concentrations than non-secretor mothers, and most HMOs differ by secretor status, but not DSLNT^37^. Lactating mothers that express FUT3 are Lewis-positive, and their milk contains 3’-FL and LNFP II. Lewis-negative mothers don’t produce FUT3. Non-secretor mothers’ milk has more neutral, non-fucosylated HMOs due to a lack of FUT2^40^. Cheema *et al*.^28^ found that human milk samples were dominated by five HMOs: 2′-FL, 3’-FL, LNT, DFLNT, and LNFP II. The secretor mothers exhibited larger amounts of 2′-FL, DFLac, LNnT, LNFP I, DFLNT, and LSTc, whereas non-secretors had higher concentrations of 3FL, LNFPII, LNT, LSTb, DFLNH, and FDSLNH^28^. Se and Le gene mutations alter FUT2 and FUT3 enzyme production, modifying the HMO structure^14^.

The most important variations within HMO distribution are the amount of fucosylated HMOs, which are prominent in secretor individuals^44^. Although the genetic profile of the mother was found to have a significant effect on the HMO composition in the mother’s breast milk, particularly fucosylated HMOs, the stage of lactation is a major determinant of the HMO quantity, and epigenetics may also have a significant effect on the HMOs’ expression^4,20,30^.

HMO concentrations and profiles vary geographically. Among healthy breastfeeding women of 11 different nationalities, McGuire *et al*.^43^ found that the concentration of 3’-FL was at least four times higher in milk collected in Sweden than in milk collected in rural Gambia, while the concentration of DSLNT was about four times lower in Sweden than in rural Gambia. Furthermore, in Gambia, lactating mothers produce considerably less HMOs (LNnT) during the wet than during the dry season^45^.

Additional maternal and environmental variables contribute to HMO variability, although their impact may be modest^16^. It was reported that after a cesarean section, human milk had lower levels of 3′-SL, 2′-FL, and 6′-GL than after vaginal delivery^32^. Parity affects as well the concentration of HMOs^28^. While parity was found to be negatively associated with LNFP III in non-secretor mothers, it was found to be positively associated with LNFP II and FDSLNH in both secretor and non-secretor mothers^28^. It is likely that parity affects HMO content due to the correlation between maternal body mass index (BMI) and human milk fatty acid composition as well as fat and protein concentration, which increases with each additional delivery^28^. Regarding the effects of prematurity on HMOs, higher levels of 3′-SL, 6′-SL, LNT, and LNDFH-I were detected in maternal milk after preterm than after term delivery. At the same time, the proportions of 3′-SL and 6′-SL also differed considerably according to the milk maturation stage^46–48^. FUT2-dependent HMOs like 2’-FL and LNFP I are slightly lower in early milk of mothers who delivered preterm^28^. But again, as stated before, it is not known if these variations in HMO concentration do have a major clinical impact.

Maternal adiposity has been reported to be positively, negatively or not related to the amount of individual and/or total HMO concentrations. Maternal body composition was shown to be related to human milk microbiota, HMO composition, and newborn body composition^28^. Maternal obesity was associated with lower concentrations of several fucosylated and sialylated HMOs. Infants born to obese mothers had reduced intakes of numerous fucosylated and sialylated HMOs, and obesity in mothers was associated with lower concentrations of these HMOs^49^. Milk from mothers who were overweight before pregnancy had higher concentrations of LNT and LNnT than milk from mothers who had a normal weight^50^. Only among secretor mothers has pre-pregnancy BMI been found to have a positive correlation with both 2′-FL and DFLac^51^. Depending on maternal secretor status, correlations between maternal weight, BMI, and body composition measurements and 2′-FL and LNH concentrations varied^28^. Adiposity measurements were positively associated with 2′-FL and FLNH concentrations in secretor and non-secretor mothers, and with 3′-SL concentrations in non-secretors^28^. McGuire *et al*.^43^ also showed a positive correlation between maternal weight and 2′-FL and BMI, but not LNH, FLNH, and FLNH. They also discovered a positive correlation between weight and LNFP III and DFLNT, and a negative correlation between weight and BMI and LNnT and DSLNT. Selma-Royo *et al*.^52^ found no connection between maternal BMI and either individual HMO profiles or clusters of HMOs. Secretor mothers have a greater dietary effect on HMO profiles than non-secretor mothers. Dietary fibers, polyphenols, and several insoluble polysaccharides, pectin, and MUFA are associated with the secretor HMO profiles. However, Plows *et al*.^33^ found that increases in HMOs over the course of 24 months of lactation were unaffected by maternal age, BMI or socioeconomic level. In Norwegian mothers, no difference in HMO composition was reported between vegan, vegetarian, and non-vegetarian mothers^53^.

In summary: HMO composition is influenced by many variables, including genetic background, environment, dietary intake, and many other factors. However, except for secretor versus non-secretor mothers, there is little evidence that these changes are of clinical impact.

## HMOs and anthropometry

There is limited information about how HMOs affect infant body composition. Total HMO intake is not related with growth and adiposity, although some specific HMOs are related with infant growth in the first six months. The difference in weight between breastfed newborns of secretor and non-secretor women may be explained in part by the fact that several HMOs are both positively and adversely linked with baby food responsiveness^4,28,48,54^. A narrative review reported that several observational studies have investigated if a link could be found between HMOs and infant growth in term-born breastfed infants^4^. Only few relationships were consistently reported across studies^4^. FLNH, LNnT, and LNFP III were negatively associated with infant anthropometric measurements and body composition, while DFLNH was positively associated^4^.

Cheema *et a*l^28^ demonstrated that anthropometrics, fat-free mass, and adiposity are all strongly linked with HMO intake, with correlations modulated by secretor status. Certain HMOs, such DFLNH and LNnT, appear to serve a protective role by controlling fat formation, perhaps protecting newborns from later-life obesity^28^. Regardless of maternal secretor status, child body composition was positively associated with 2′-FL, 3-FL, DFLac, DFLNH, DFLNT, and LSTb intakes^28^.

In infants of non-secretor mothers, DFLNT concentrations were positively- and FLNH, 6′-SL, and FDSLNH were negatively associated with infant anthropometric measurements and body composition^28^.

In infants born from secretor mothers, 3′-SL intake was linked to weight, length, fat-free mass, and weight for age^28^. 3‘SL was the only HMO linked with greater weight for length increases in the first four months of lactation in a recent European multicenter study of 370 mother-infant dyads^55^. Still in secretor mothers, HMO composition at three months after birth was linked to weight and height during the first five years of life^51,56^. An inverse relationship between HMO diversity and LNnT concentration and a direct relationship between 2′-FL concentration and z-scores was reported for children’s height and weight z-scores^51^. However, other studies reported different: a negative correlation between LNnT and food responsiveness in the first month of life, but DFLNT and DSLNT showed this correlation solely among secretors^54^. Positive associations were seen between DSLNH, FLNH, LNH, LSTc, and food responsiveness at 6 months in both the overall population and in secretors exclusively^54^.

In a Gambian study, researchers found that different HMOs, and 3’-SL in particular, affected infants’ weight-for-age z scores, whereas relative sialylation of HMOs did not^45^. Infants receiving higher total HMO concentrations had higher percentages of fat-free mass and a lower fat-to-fat-free mass ratio and fat-free mass-to-fat-mass ratios^57^. Alderete *et al*.^58^ showed that lower infant weight at one and six months, as well as reduced lean and fat mass at six months, were associated with higher levels of LNFP1 and a positive correlation was observed with greater fat mass and LNFP-II and DSLNT.

In 2016, two cohorts of mothers in Malawi, one in healthy 6-month-old, and another in severely stunted infants, HMO compositions were studied^59^. Breast milk of undernourished infants has lower levels of HMOs than the milk that healthy babies received^59^. Among secreting mothers, there was no difference in HMO concentrations between those infants who were healthy or stunted. But milk from non-secretor mothers of stunted infants had lower levels of fucosylated and sialylated HMOs than infants with normal growth^60^. These results suggest that milk of non-secretor mothers would be less conducive to child growth due to an inefficient compensation for a lack of fucosylated HMOs^48,60^.

According to data from Bangladesh, there is an increased likelihood of severe acute malnutrition for every unit increase in the relative abundance of sialylated HMOs^61^. Fifty-four percent of the infants with severe acute malnutrition and 58% of the infants who were not malnourished were born to women who were secretors. Fucosylated or undecorated HMOs were not shown to be significantly linked to severe acute malnutrition. This suggests that human milk with a higher relative abundance of sialylated HMOs might have a detrimental effect on the nutritional health of children under the age of^61^.

Two hypotheses may be related to the plausibility of HMOs on anthropometric measurements: i) certain HMO-microbiota pairs may affect infant anthropometry, and ii) HMOs affect food-responsiveness and appetite via a microbiome-driven process that affects the entero-endocrine system or the central nervous system^4^. The developing gut microbiome is regarded as a crucial determinant determining infant growth, along with the environment, genes, epigenetics, and metabolism^62^. Sprenger *et al*.^4^ hypothesize that differences in maternal nutritional status and in the composition of the mother’s gut microbiota (including epigenetic and genetic changes) may be significant confounding variables. Randomized controlled trials (RCTs) and mechanistic studies are needed to show if the inclusion of specific HMOs could aid to promote growth in specific circumstances of faltering growth or in preterm-born infants.

### HMOs and microbiota

Gut microbiota composition is established in early life and is influenced by many variables, such as delivery mode, gestational age, maternal, and infant/toddler nutrition, antibiotic use, presence of siblings, local environment, geographic location, and host genetics has short- and long-lasting effects on health^63^. The content of HMOs in mother’s milk is one of the variables determining the composition of the gut microbiota in the infant^63^. Infant microbiota is characterized primarily by low diversity and high variability, even more than in adults^64^. Breastfed infants have a significantly different microbiota and metabolome compared to formula-fed ones^65^. Bifidobacteria are among the first colonizers of the infant gut and sustaining this abundance of Bifidobacteria is crucial to preserving the gut microbiota composition. Several studies have shown that HMOs influence the gut microbiota composition via bifidogenic and anti-pathogenic effects and by potentially interacting with the gut epithelium to alter the physical interactions between microbes and their hosts^66^. Breastfeeding, due to the supply of HMOs into the gut, promotes the growth of specific HMO-utilizing *Bifidobacterium* species which are nearly accounting for 50–90% of the total bacterial population found in the feces of breastfed newborns^67^. In the first 1000 days of life, the gut microbiota of healthy breastfed infants is typically dominated by ‘infant-type’ bifidobacteria, including *Bifidobacterium longum* subsp. *Infantis*, *B. bifidum*, *B. breve*, and *B. longum* subsp. *longum*^68^. Some members of the *Bifidobacterium* genus can metabolize HMOs, but not all of them can, and not all HMOs cause the same changes in the composition and/or activity of the gut microbiota and have the same effects on host well-being and health. *B. longum* subsp. *Infantis* is the most effective consumer of HMOs, and *B. bifidum* and *B. breve* can also partially consume HMOs^20^. *Bifidobacterium bifidum* and *B. longum subsp. infantis*, two avid HMO consumers, dominate through inhibitory effects in which the early arriving species apparently depletes resources for later arriving species^69^. *Bifidobacterium longum* would be a moderate competitor, as it cannot consume LNnT, but can consume LNT and specific fucosylated sugars such as 2′-FL, 3-FL, LDFT, and LNFP I. *Bifidobacterium breve*, a species with limited HMO-utilization ability, limited to LNT and LNnT, can benefit from facilitative priority effects and dominates by utilizing fucose, an HMO degradant not utilized by the other bifidobacterial species like *B. bifidum* and *B. infantis*^69^. Several *Bacteroides* species are known to utilize HMOs as well. *Bacteroides* have been reported to dominate in the absence of bifidobacteria, and mutual exclusion may be occurring through the depletion of HMOs^68^. *Bacteroides thetaiotamicron*, found in a healthy mature gut, provides metabolic and immune support and is an effective HMO degrader^70^.

The diversity of bifidobacteria in is closely correlated with whether or not the mother is a secretor for the enzyme FUT2^71^. Observational studies showed that secretor milk status (due to its high levels of 2′-FL and other Fucosyl-HMOs) are associated with bifidobacteria dominated early gut microbiota in breastfed infants^43,72,73^. Stool from infants with a microbiome harboring this 2‘FL utilizing capacity has been shown to have a lower pH and provides better protection against specific diarrheal diseases^73^. Bifidobacteria isolated from the stool of secretor breast milk-fed infants were able to utilize 2′-FL as the sole carbon source, indicating a more pronounced bifidobacterial metabolic activity targeting fucosylated HMOs^4,74^. Conversely, the gut microbiota of infants born to non-secretor mothers is depleted of bifidobacteria because to the absence of 2′-FL in human milk, which may result in a diminished level of biological defense against infections^34^. In contrast, bifidobacteria colonization is slowed by non-secretor human milk, while *Clostridium* and *Enterobacteriaceae* are encouraged^73^.

When HMOs are fermented by bacteria, SCFAs are produced, creating a low-pH environment in the colon that encourages the growth of beneficial bacteria and inhibits pathogens^20,75^. These SCFAs have multiple beneficial physiological effects, such as acting as anti-inflammatory agents, serving as energy substrates for intestinal epithelial cells, and promoting gastrointestinal motility^6,76^. Cross-feeding (when one kind of bacterium’s metabolic byproducts are used as a food source by another type of bacterium in the environment) is encouraged by the presence of HMOs^77,78^. The bifidobacterial population in the infant’s gut is composed of a co-group of multiple *Bifidobacterium* strains, rather than one strain dominating, and competing to the exclusion of all others. On the one hand, the cross-feeding effect among bifidobacterial species/strains is associated with the ability to thrive in HMOs of multiple *Bifidobacterium* members in the infant’s gut. Fermentation products of HMO-degrading infant-type *Bifidobacterium* species may suppress other gut microbes and opportunistic pathogens that do not use HMOs. This competitive advantage in the HMO use of the developing gastrointestinal tract greatly affects the survival and persistence of beneficial *Bifidobacterium* species and lessens the burden of potentially harmful or pathogenic bacteria^6,68^. On the other hand, certain bifidobacterial taxa cooperate with non-bifidobacterial taxa (including HMO consumers and non-HMO consumers) to maximize the nutrient consumption of HMOs, thus contributing to increased bifidobacterial diversity and dominance-gaining^68^. Schwab *et al*.^79^ showed that *Eubacterium hallii* consumes the fermentation products of HMO by bifidobacteria and generates butyrate and propionate. The cooperation of the bacterial community in the neonatal intestine to maximize the utilization of HMOs, so as to maintain the intestinal immune balance of newborns. Overall, infant-type *Bifidobacterium* species are well adapted to the infant gut and efficiently consume HMOs, and their presence influences both immediate and long-term health outcomes^68,80^. Since HMO composition differs between mothers, it’s reasonable to assume that each mother’s milk has a unique effect on her infant’s gut microbiota.

In addition to the widespread indirect effects resulting from microbial fermentation of HMOs, recent research has described the direct benefits of HMOs on gut health^6^. 3´-FL stimulated production of mucin and antimicrobial peptides in goblet cells, and 2′-FL may have a similar effect on goblet cell function when inflammatory stressors are also present^81^. Natividad *et al*.^82^ used in vitro models that replicate the microbial ecology and the intestinal epithelium to evaluate the impact of lactose, 2’-FL, 2’-FL + LNnT, and a mixture of six HMOs (2’-FL, LNnT, DFLac, LNT, 3’-SL, and 6’−SL) on newborn gut microbiota and intestinal barrier integrity. Although the SCFA levels were higher and bifidogenic potential was present in all the products examined, only the fermented medium from the HMOs provided protection against inflammatory gut barrier disruption. The most butyrate-producing bacteria were enriched by the six HMOs formulation, whereas 2’-FL/LNnT and six HMOs promoted the greatest diversity within the *Bifidobactericeae* family^82^.

Since the intestinal epithelial glycocalyx is crucial for microbial colonization, Kong *et al*.^83^ conducted the first study to examine the development of this barrier in relation to HMOs. They found that 2′-FL and 3-FL stimulate glycocalyx formation and have a direct effect on the growth of epithelial cell lines. HMOs have been proven to directly modulate goblet cells, causing them to produce more mucus, another important component of the intestinal barrier system^81^.

There is a limited information on the complicated relationships between the human milk microbiome and different types of HMOs^5,28^. Although the potential biological influence on the newborn is still unclear, there is an association between maternal secretor status and HMOs with human milk microbiota^71^. Maternal factors including body composition are related to human milk microbiota and HMO composition. Individual HMO concentrations may influence human milk bacterial profiles during the exclusive breastfeeding period. Total HMOs and 2′-FL were positively associated with the relative amount of *Staphylococcus*, whereas 3′-SL was negatively correlated with the proportions of *Ralstonia* and *Novosphingobium* in 16 human milk samples^84^. *Staphylococcus epidermidis, Streptococcus salivarius, Cutibacterium acnes, Gemella haemolysans*, and *Veillonella nakazawae* all had correlations (positive and negative) with HMO concentrations^28^. In colostrum, a higher total HMO concentration is associated with higher counts of *Bifidobacteria*. Sialylated HMOs were positively correlated with *B. breve*, and non-fucosylated/non-sialylated HMOs were positively correlated with *B. longum*. There were also favorable associations found between fucosylated HMOs and *Akkermansia muciniphila* and between fucosylated/sialylated HMOs and *Staphylococcus aureus*^85^. Only in non-secretor mothers, several HMOs were correlated negatively with *Streptococcus parasanguis, Gemella haemolysans*, and *Cutibacterium* acnes. Among the secretor mothers, 3′-SL was negatively associated with *Staphylococcus epidermidis*. Moossavi *et al*.^86^ found that 3′-SL, 6′-SL, LSTb, LSTc, DSLNT, and DSLNH all have positive relationships with *Staphylococcus spp*.

HMOs are the third most important component of human milk and are crucial for the development of a healthy early life gut microbiome. As a result, it is evident that HMOs encourage the growth of a bifidobacteria-rich gut microbiome.

### HMOs and necrotizing enterocolitis (NEC)

In preterm newborns, breastfeeding has been linked to a lower incidence of NEC compared to formula feeding^4,87,88^. In a murine model of NEC, HMOs raise mucin levels and lower bacterial attachment^89^. FUT-2 non-secretor and low secretor status in premature newborns is associated with a higher risk for NEC, gram-negative sepsis, and death^90^. HMO diversity and specifically DSLNT were shown in observational studies to be associated with NEC^4,87,91–93^. Although explanations for the association between DSLNT and NEC remain elusive, an age-appropriate microbiome progression was suggested^91^. DSLNT was shown to increase survival rate and reduce pathology scores in a rat model of NEC^94^. More studies are needed to understand the link between DSLNT and NEC risk.

Protective effects against (severity of) NEC were observed for 6’−SL and 2’-FL in experimental models^87,94–96^. Both 2’−FL and 6’-SL suppress toll like receptor-4 activation, which is linked to the onset of NEC, and hence decrease inflammation in mouse and piglet models of NEC^95^. However, clinical observations could not confirm a relation between 2′-FL or 6’−SL with NEC risk.

### HMO and infections

In, the amount of HMOs is associated with a decreased prevalence of diarrhea, overall infections, and morbidity^97–100^. FUT2 alleles are associated with a higher risk of infant gastrointestinal and respiratory illnesses^101^. At the age of 2 years, diarrhea due to stable toxin-*Escherichia coli* infection and of unknown etiology were both reduced in breastfed infants with high levels of alpha 1,2-linked fucosylated-HMOs^102^. Higher levels of LNFP-II in colostrum were associated with reduced respiratory and gastrointestinal infections by 6 and 12 weeks^98^. Torres-Roldan *et al*.^110^ investigated the HMOs’ composition and infection rates in very-low-birth-weight infants, FDSLNH was found to protect for late-onset neonatal sepsis^103^.

In breastfed newborns in Mexico, the incidence of Campylobacter diarrhea was decreased in infants whose mothers’ milk had a high percentage of 2′-FL^97^. The protection offered by HMOs was limited to the duration of breastfeeding^105^. Furthermore, high levels of LNDFH-I, another 2-linked fucosyloligaosaccharide, protect against calicivirus diarrhea including norovirus^97^. Population studies show significantly higher levels of LNnT, 2’−FL and 6’-SL in milk of mothers of rotavirus-positive neonates with gastrointestinal symptoms^104^. However, it is unknown whether high levels of these HMOs are a natural reaction to the rotavirus infection or whether they provide poorer protection against a rotavirus infection than lower levels^104^. Secretor-positive human milk inhibits norovirus particles, while secretor-negative milk does not, suggesting that alpha 1,2 linked fucosylated-HMOs may be implicated^105^. Both 3-FL and 2’-FL have been found to bind norovirus^33^.

Higher concentrations of LNF-II in human milk at two weeks postpartum were associated with fewer respiratory problems in infants by 6 and 12 weeks of age^106^. Mother’s milk of sick infants contains more of certain HMOs (LNT) than healthy infants, while other HMOs (LNFP1) are less frequent in sick infants^45^. However, the levels of HMOs could not be related to physician reported data on infections (otitis media, upper and lower respiratory tract infections)^107^.

HIV-infected women have larger relative abundances of 3’-SL in their milk than HIV-negative mothers^108^. HIV-infected women with total HMOs above the median (1.87 g/L) are less likely to transmit HIV via breastfeeding, although there was no difference related to secretor or Lewis status^109^. A higher LNnT concentration correlated with reduced transmission. Independent of other known risk factors, higher concentrations of non-3’-SL HMOs were associated with decreased likelihood of postnatal HIV transmission. In Zambian children, breastfeeding was protective against mortality only in uninfected children with high concentrations of fucosylated HMOs^110^. Higher amounts of 2’-FL and LNFP I, as well as 3-FL and LNFP II/III, were substantially associated with a decreased mortality in children who were not HIV-infected^110^. Breastfeeding was found to reduce mortality risk for HIV-infected children, but no consistent relationships were found between HMOs and mortality^110^.

Some potential modes of action for HMOs include weakening, preventing, and deviating pathogens from adhering to their cognate cell surface ligands^6^. Several viruses and bacteria have been found to bind to HMOs^4^. Many infectious agents, including viruses (including influenza virus, respiratory syncytial virus, coronaviruses, rotavirus, HIV, and norovirus), bacteria (including *Streptococcus pneumoniae, Haemophilus influenza*, Group B streptococci (GBS)), and protozoan parasites, require adhesion to the surface of epithelial cells in order to replicate and, in some cases, infiltrate and cause disease^80,111^. HMOs act as soluble decoy receptors that block the attachment of specific viral, bacterial, or protozoan parasite pathogens to the epithelial cell surface^117^. Pathogens that are not bound to the cell surface are washed away harmlessly. Animal models have indicated that increasing acetate, in combination with other metabolites, increases protection from gastrointestinal and respiratory infections^112,113^.

Regarding to anti-infective properties of HMOs, studies showed^6,80,114,115^

- **2’-FL**: C. *jejuni, Enteropathogenic E. coli, Salmonella enterica*, rotavirus, norovirus, respiratory syncytial virus
- **3’-FL**: *Enteropathogenic E. coli, Salmonella enterica*, norovirus,
- **LNT**: Vibrio cholerae toxin, Group B streptococcus, *Entamoeba histolytica*
- **3’-SL**: *Enteropathogenic E. coli*, Vibrio cholerae toxin, *Helicobacter pylori*, Pseudomonas aeruginosa, rotavirus, influenza
- **6’-SL**: *Enteropathogenic E. coli, Helicobacter pylori*, *Pseudomonas aeruginosa*, influenza A H1N1, rotavirus
- **LNnT**: pneumococci, influenza

Some HMOs are bacteriostatic against GBS, causing neonatal sepsis, pneumonia, and meningitis^87^. Non-sialylated HMOs, LNT and LNDFH-I (1–2 mg/L daily), delay the growth of GBS with 96–98%^116^. HMOs also showed antibacterial action against, *Acinetobacter baumannii*, and *Staphylococcus aureus*^41^. In neonates, HMOs alter the growth and morphogenesis of *C. albicans*, which then makes it more difficult for the pathogen to attach, invade, and cause disease^126^.

According to basic and animal research, HMOs appear to have a role in the treatment and prevention of bacterial, viral, protozoal, and fungal diseases. It is important to note that the majority of the evidence presented in support of the anti-adhesive effects of HMOs originates from experimental studies. It will need well-designed and powered mother-infant dyad observation studies and, more crucially, intervention studies to demonstrate that a single HMO or a mixture of several HMOs reduces the incidence and/or severity of a diversity of infectious diseases.

### HMO and immune development

The immune system develops over the course of gestation and continues to be postnatal in relation to exposure of microorganisms. HMOs can modify host epithelial and immune cell responses and contribute to the development of the gastrointestinal immune system^4–6,20,48,117^. It has been hypothesized that HMOs influence the responses of epithelial cells and immune cells by modifying cell proliferation, differentiation, and apoptosis, as well as cell signaling pathways and cell surface glycosylation, so modulating immunological functions. Intestinal epithelial barrier cells can be directly affected by HMOs of varying structures. Direct interactions between HMOs and infant intestinal epithelial cells affect their gene expression, cell cycle, and cell surface glycosylation and regulate their growth, differentiation and apoptosis^20^. The establishment of the infant gut microbiota and its metabolic activity is thought to be an important mechanism through which HMOs affect immune system development^4^.

In addition, when HMOs reach the colon and are then absorbed intact into the circulation, they may play a systemic immunomodulatory role by mediating cell-cell interactions in the immune system. Intestinal health and intestinal barrier function constitute the first defense line in innate immunity^4–6,20,48,118,119^. As shown in vitro, HMOs inhibit cell proliferation, promote cell differentiation, death, and maturation, and strengthen the barrier function^7,31,94,120^. Modulations in gene expression caused by HMOs have an immediate effect on intestinal epithelial cells, altering their surface glycans and eliciting different cellular responses. The generation of cytokines by lymphocytes is altered by HMOs, which may result in a more balanced TH1/TH2 response. Growing evidence from in vitro research suggests that HMOs directly control immunological responses by altering immune cell populations and cytokine release in infants, in addition to their indirect effects on the immune system via changes in gut microbiota^94^.

HMOs may also affect immune system receptors. Galectins, glycan-binding proteins, regulate intracellular signaling, cell – cell communication, proliferation, and survival^121^. Galectins may be HMO receptors for the immune system development^5^. HMOs can act locally or systemically on mucosa-associated lymphoid cells^15^.

HMOs contain tolerogenic factors influencing human monocyte-derived dendritic cells and elevated Interleukin (IL)-10, IL-27, and IL-6 levels but not IL-12p70 and tumor necrosis factor-alpha^122^. 2′-FL increases Th1-type interferon-gamma and regulates IL-10 production, suggesting a Th1 response^123^. CD11(+) mesenteric lymph node dendritic cells exposed to 3’-SL can produce cytokines that boost Th1 and Th17 immune cells^124^. Three weeks of 2’-FL administration to Caco-2Bbe cells, reduced the permeability and upregulated tight junction proteins^125^. 2’-FL can boost innate and adaptive immunity in influenza-specific mouse models and reduce respiratory viral infections^126^. In a mouse influenza vaccination model, dietary 2′-FL improved humoral and cellular immune responses, boosting vaccine-specific delayed-type hypersensitivity and immunoglobulin proliferation.^127^.

### HMO and allergy

The prebiotic effects and the immunological programming provided by HMOs also affect individual susceptibility to allergies. A balanced microbiota and microbiome provide immunological benefits by lowering the risk of allergic disorders through the synthesis of SCFAs, such as butyrate and propionate, which have anti-inflammatory and anti-allergic qualities. It is known since more than 20 years that the gastrointestinal microbiota differs in allergic and non-allergic infants before symptoms of allergy develop^120,128,129^. A significant reduction in the probability of acquiring immunoglobulin E (IgE) mediated eczema at the age of two years was observed in C-section-born, allergy-prone breastfed infants whose mothers expressed FUT2, resulting in 2′-FL synthesis in human milk^39^. C-section infants who were administered human milk containing FUT2-dependent oligosaccharides were shown to have a lower incidence for IgE-associated eczema at the age of 2 years^39^. It was only in infants born via C-section that these associations between IgE-associated eczema and consumption of FUT2-dependent milk oligosaccharides were observed^38^. The authors did not find an association with HMOs and allergic disorders at 5 years of age^39^. When compared to milk with high LNFP III concentrations, infants who received human milk with low LNFP III concentrations were more likely to develop cow’s milk protein allergy (CMPA)^38^. The mothers’ FUT2 status was associated with a delayed onset of CMPA, and CMPA infants born to non-secretor moms (FUT2 negative) were more likely to develop IgE-mediated CMPA. Lower levels of DSLNT and 6′-SL were associated with atopic dermatitis^38^. Concentrations of nine neutral HMOs were not associated with the chance of having an allergic disease up to the age of 18 months, according to a case-control study in 20 mother-infant pairs from a larger birth cohort^97^.

Regarding to relationship between food sensitization, a large clinical observation study (421 mother – infant dyads) demonstrates that HMO composition is associated with the development of food sensitization^130^. The HMO profiles associated with lower risk of food sensitization were characterized by higher concentrations of FDSLNH, LNFP II, LNnT, LNFP I, LSTc and FLNH, and lower concentrations of LNH, LNT, 2′-FL, and DSLNH^130^. In an ovalbumin sensitized mouse model, 2’-FL and 6-FL stabilize mast cells by inducing expression of T regulatory cells and activate the IL-10(+) regulatory cells to reduced symptoms of food allergy^131^.

By influencing the colonization of the gut microbiota and producing butyrate, microbiota composition of human milk helps the prevention of development of food allergies^50^. The development of a microbiome dominated by bifidobacteria was significantly delayed in infants fed secretor-negative human milk compared to those fed secretor-positive breastmilk at three months of age^132^. In particular, *B. breve* has been linked to a decreased incidence of eczema^133^. Among infants with a family history of atopy, reduced *Bifidobacteriaceae* abundance in infancy is related with a higher risk of eczema^133^. However, another study found no significant association between the intake of particular HMOs (measured at 6 weeks and 6 months) and the risk of atopic dermatitis^134^.

Breastfeeding has been shown to reduce the likelihood of developing food allergy, eczema, and asthma, at least during early life, although there is a lack of consistency in reporting of breastfeeding duration, diagnostic criteria for atopic dermatitis, and assessment age^135^.

### HMO and brain/cognitive development

Sialic acid is considered a key conditioned nutrient during early development. Although the mechanisms are not completely understood, the high levels of sialic acid in human milk, especially in the form of sialylated milk oligosaccharides, are considered an important bioactive component linked to infant brain and cognitive development^4,6,136^. Both 3’−SL and 6’-SL have been shown to enhance learning and memory and play a role in the gut microbiota-brain axis^137–139^.

Cho et al.^149^ showed that the association between human milk 3’-SL concentration and cognition, particularly language functions, in typically children who received human milk containing alpha tetrasaccharide (an HMO, which only be detected in the mothers with blood type A. High levels of 6′-SL have been linked to better cognitive and motor development at 18 months of age, as well as better language development at 12 months of age^140,141^.

In the brain, fucosylated proteins are found along the neuronal synapses, particularly in the hippocampus, where they play a crucial role in the development of memory and learning^142^. There is experimental evidence that 2’-FL interferes with cognitive processes, including enhanced cognitive ability, learning, and memory^143^. Early exposure to 2′-FL and 6`-SL represents a critical time window for the positive influence on the cognitive development at 2 years^48,140,141^. Although human data are scant, one study found that breastfed infants with greater 2’-FL intake at one month of birth had better cognitive development at 24 months of age and improved motor skills^144,145^. A higher concentration of fucosylated HMOs was linked to better linguistic development between the ages of 12 and 18 months^140^.

In summary, studies suggest a role of HMOs in brain and cognitive development, but more data are needed. The mechanisms of action need to be further unraveled.

### HMO and diabetes

2’-FL, 3’-SL, 6-SL, and LNnT may have protective effects on the development of type-1 diabetes. In an animal model, early life intake of HMOs delayed and suppressed type-1 diabetes development in non-obese diabetic mice and reduced the development of severe pancreatic insulitis in later life^126^.

### HMO and infant formula

#### Effects of HMOs containing infant formula on anthropometry

Although the WHO recommends exclusive breastfeeding since birth to 6 months of age, some infants will not receive human milk. The energy and nutrition need of a growing infant can be met by infant formula, which typically is cow’s milk based. However, cows and human milk differ substantially in the composition of macro- and micro-nutrients, and in the content of bioactive components^26^. In fact, HMOs are virtually absent in cow’s milk (or any animal milk), and their variety is much lower than in human milk^146^. Observational studies revealed that many disorders such as NEC, irritable bowel syndrome, obesity, allergies, and eczema, are more common in formula-fed compared to breastfed infants^20^. The early microbiota development and effect on immune system development in cow’s milk formula fed infants might be affected by the lack of HMOs^147^. Nowadays, it is possible to supplement infant formula with mixtures of HMOs. The effects of HMOs in infant formula have been evaluated in several randomized clinical trials (Table 2).

**Table 2.** HMOs in formula in research.

Reference	HMO	Study site	Inclusion criteria	Intervention	Control group(s)	Study duration	Primary outcome	Secondary outcomes: Test *versus* control
*Puccio et al.*^*118*^ *2017*	2‘FL + LNnT	Belgium, Italy	0 to 14 days	0–6 months: Starter formula + 1.0 g/L 2‘FL + 0.5 g/LNnT 6–12 months Follow-up formula	l0–6 months Starter formula 6–12 months Follow-up formula Starter formula: Intact protein, cow’s milk – based, whey-predominant infant formula with long-chain polyunsaturated fatty acid.	12 months	Weight gain: similar Mean difference [95% CI] test vs control: −0.30 [−1.94, 1.34] g/day	The formula with 2′-FL and LNnT was well-tolerated. The formula with 2′-FL and LNnT supported normal, age-appropriate infant growth during the 4-month exclusive feeding period, after the introduction of complementary foods from 4 to 6 months, and following the switch to a standard follow-up formula without HMOs at 6 months up to 12 months of age. Infants receiving formula with 2′-FL and LNnT had significantly softer stools and fewer episodes of nighttime wake-ups at age two months, and infants born by cesarean section also had a lower incidence of colic at four months of age. Infants receiving HMO containing formula had significantly fewer parental reports of parent-reported morbidities related to lower respiratory tract infections as well as antipyretic and antibiotic.
*Alliet et al.*^159^ *2022*	2’FL + L. Reuteri DSM 17,938	Belgium, Italy 2017–2019	Healthy, term (37–42 weeks gestation) infants aged≤14 days at enrollment with a birth weight between 2500 and 4500 g	Standard bovine milk-based whey predominant formula with an energy density of 670 kcal/L containing 75 g/L lactose, 34 g/L fat, 14 g/L protein (60:40 whey: casein ratio), and *L. reuteri* DSM 17,938 + 1 g/L 2’FL which i	Standard bovine milk-based whey predominant formula with an energy density of 670 kcal/L containing 75 g/L lactose, 34 g/L fat, 14 g/L protein (60:40 whey:casein ratio), and *L. reuteri* DSM 17,938 Breastfed infants	180 days	Weight gain in HMO formula was non-inferior to a non-HMO supplemented formula.	Anthropometric Z-scores, parent-reported stooling characteristics, gastrointestinal symptoms and associated behaviors, and AEs were comparable between formula groups. The microbiota composition in formula containing 2’-FL and *L. reuteri* DSM 17,938 approaching BF. At age 1 month, *Clostridioides difficile* counts were significantly lower in study group than control. *Bifidobacterium* relative abundance in study fromula tracked toward that in breast-fed.
*Marriage et al.*^*149*^*2015*	2‘FL + GOS	United States 2013–2014	Healthy, singleton infants (birth weight≥2490 g), who were enrolled by day of life 5	Test formula 1: formula with 2.4 g/l GOS Test formula 2: formula with 2.4 g/l GOS+0.2 g/l 2’FL Test formula 3: formula with 2.4 g/l GOS+1 g/l 2’FL	Breastfed infants	4 months	No significant differences among feeding groups for weight, length or head circumference gain during the 4-month study period (*p* = 0.016 from day 14–28 and *p* = 0.022 from day 84–119).	2’FL was found in the plasma and urine of infants fed a 2’FL formula, no significant differences were seen in 2’FL uptake relative to concentration fed (no *p* value mentioned).
*Goehring et al.*^165^*2016*	2‘FL + GOS	United States	Healthy singleton infants (birth weight≥2490 g) who were enrolled by 5 days	Test formula 1: formula with 2.4 g/l GOS Test formula 2: formula with 2.4 g/l GOS+0.2 g/l 2’FL Test formula 3: formula with 2.4 g/l GOS+1 g/l 2’FL	Breastfed infants	4 months	Breastfed infants and infants fed experimental formulas with added 2’FL were not different. They had 29%-83% lower concentrations of plasma inflammatory cytokines than infants fed the formula with only GOS added.	Peripheral blood mononuclear cells were not different in breastfed infants compared to the infants fed a 2’FL supplemented formula. However, they had significantly lower concentrations of TNF-α (*p* ≤ 0.05), interferon γ (*p* ≤ 0.05) than infants fed the GOS-only formula.
*Parshat et al.*^*150*^*2021*	2’FL, 3-FL, LNT, 3’-SL and 6’-SL.	Germany, Italy, and Spain 2018 × 2020.	Healthy f infants≤14 days of age, born at full term (≥37 and≤42 weeks of gestational age), singleton birth, with a birth weight of 2500–4500 g and an APGAR score of 9 or 10 as assessed within the first 15 min after birth. (*n* = 341)	5.75 g/L of a mix of 2’FL, 3-FL, LNT, 3’-SL and 6’-SL.	Infant formula without HMOs Breastfed infants	4 months	No significant difference in weight gain between both formula groups (*p* < 0.001).	No difference in length or head circumference gain between both formula groups. The test formula was well tolerated. Test group: softer stools at higher stool frequency than control formula group. Adverse events: similar in all groups.
*Lasekan et al.*^*152*^*2022*	2’FL, 3-FL, LNT, 3’-SL and 6’-SL.	United States September 2019 through December 2020, mostly during the COVID-19 pandemic	Singleton, healthy term infants (gestational age 37–42 weeks) between 0 and 14 days of age at enrollment, with a birth weight≥2490 g. (*n* = 222)	5.75 g/L of a mix of 2’-FL, 3-FL, LNT, 2’-SL and 6’-SL.	Infant formula without HMOs Breastfed infants	4 months	The primary outcome for this study was weight gain per day, from D14 to D119: No significant differences in weight gain (*p* ≥ 0.337).	Secondary variables were weight, interval weight gain per day, length, interval length gain per day, head circumference and interval HC gain per day. No significant differences among the three groups regarding gains in weight and length (*p* ≥ 0.05). More soft, frequent and yellow stools in the test group in comparison to the standard milk-based formula group. This makes the stools more similar to breastfed infants. Parental responses indicated that the test group had a higher average loose stool dimension score compared with control at D119 (all *p* ≤ 0.033). The standard milk-based formula group saw significantly more frequently (*p* = 0.044) healthcare professionals for illness in comparison to the test group.
*Estorninos et al.*^*164*^ *2022*	MilkOligoSacch-arides (MOS)	Phillipines 2016–2018	Healthy term, singleton birth (37–42 weeks of gestation), postnatal age of 21–26 d at enrollment, *3*) weight-for-length and head circumference-for-age *z*-scores between −3 and+3 and n = 230	Control formula with MOS ingredient total of 7.2 g oligosaccharides per liter.	Breastfed infants Control formula: 65% intact whey protein (enriched in α-lactalbumin) and 35% casein protein ratio, carbohydrates consisting of 100% lactose, and a vegetable oil blend high in *sn-2* palmitate.	4 months	Overall microbiota composition in test group was different from standard formula group (*p* < 0.05).	*Bifidobacterial* abundance was higher (*p* < 0.05) in test group compared to standard formula group, approaching breastfed infants. Significantly lower number of *Clostridioides difficile* (*p* < 0.001) and *Clostridium perfringens* (*p* < 0.01) in test group compared to standard formula group. Fecal secretory IgA in test group was significantly higher (*p* < 0.001) compared to standard formula group and closer to breastfed infants. Test group and breastfed infants had significant lower fecal calcium excretion (*p* < 0.005) and fecal pH (*p* < 0.001) and higher lactate (*p* < 0.001) compared to standard formula group.
*Bosheva et al.*^*25*^ *2022*	2’-FL, DFL, LNT, 3’-SL, 6’-SL	Bulgaria, Hungary, and Poland 2018 and November 2021	Healthy and full-term, with birth weight between 2,500 and 4,500 g, and aged≥7– ≤21 days at enrollment. *N* = 50	Test formula 1: 1.5 g/L of a mix of 2’-FL, DFL, LNT, 3’-SL, 6’-SL Test formula 2: 2.5 g/L of a mix of 2’-FL, DFL, LNT, 3’-SL, 6’-SL	Standard cow’s milk-based formula Breastfed infants	15 months	Microbiota in the two test groups were significantly (*p* < 0.01) different in comparison to the standard cow’s milk formula group (SFG).	Significantly higher abundance of *Bifidobacterium longum subsp. Infantis* (*p* < 0.05) in test group in comparison to SFG. Significantly (*p* < 0.05) lower number of *Clostridioides difficile* in test group in comparison to SFG and comparable to breastfed infants. Higher secretory immunoglobulin A and lower alpha-1-antitrypsin (*p* < 0.05) in the test group in comparison to SFG.
*Vandenplas et al.*^*151*^ *2020*	2’-FL, 3’-GL, scGOS/lcFOS	Belgium, Hungary, Poland, Spain Ukraine	215 fully formula fed infants≤14 days old	Infant formula contained 26% fermented formula with postbiotics derived from the Lactofidus fermentation process (including 3′-GL), 0.8 g/100 mL scGOS/lcFOS (9:1), and 0.1 g/100 mL 2′-FL	Standard infant formula containing the oligosaccharides scGOS/lcFOS (0.8 g/100 mL; 9:1), but no 2′-FL, postbiotics, or milk fat. Breastfed infants	Until 17 weeks of age	Equivalence in weight gain between the test formula and control infant formula up to 17 weeks of age	Supported an adequate growth, was well-tolerated, and no safety concerns were revealed given the absence of clinically relevant differences in the number adverse event.s
Nowak-Wegrzyn et al. ^168^ 2019	2‘FL + LNnT	United States 2017–2018	64 children with CMPA Aged 2 months to 4 years	Extensively hydrolyzed formula with 1 g/L 2’-FL and 0.5 g/L LNnT	Extensively hydrolyzed formula without HMOs	7–9 days	The study formula met the clinical hypoallergenicity criteria.	
Vandenplas et al. ^169^ 2022	2‘FL + LNnT	Poland, Italy, United Kingdom, Spain, Hungary, Belgium, Singapore February 2017 and August 2018	Full-term infants aged 0–6 months with physician-diagnosed CMPA *N* = 194	100% whey-based EHF supplemented with 2′-FL at a concentration of 1.0 g/L and LNnT at 0.5 g/L	100% whey-based extensively hydrolyzed formula without HMOs	4 months	Daily weight gain with the test formula was noninferior to the formula without HMOs (*p* < 0.005).	No significant group differences in anthropometric parameters. The formula was tolerated well, and the safety profiles of the test and control formulas were similar Highly significant reduction in CMPA symptoms, as evidenced by a fall in CoMiSS to levels reported in healthy infants test formula. Showed significant reduction in frequency of upper respiratory tract infections (*p* = 0.003) and otitis media (*p* = 0.045) compared to the control formula.
Berger et al. ^161^ 2020	2‘FL + LNnT		Follow-up study Puccio et al.	0–6 months: Starter formula + 1.0 g/L 2‘FL + 0.5 g/LNnT 6–12 months Follow-up formula	0–6 months Starter formula 6–12 months Follow-up formula Starter formula: Intact protein, cow’s milk – based, whey-predominant infant formula with long-chain polyunsaturated fatty acid.			The microbiota of formula-fed 3-month-old infants was different if they received HMOs and closer to the microbiota of BF infants. This was observed for the microbial diversity, the global composition at the genus level, and the abundance of several major genera typical of that age period. An increase of bifidobacteria, a decrease of *Escherichia* and unclassified *Peptostreptococcaceae*, a family to which *Clostridium difficile* belongs
Gold et al. ^154^ 2022	2‘FL + LNnT	Australia 2018–2021	Term infants aged 1–8 months with physician-diagnosed moderate-to-severe CMPA	amino acid-based formula (AAF) with 2′-FL and LNnT, at concentrations of 1.0 g/L and 0.5 g/L	Amino acid-based formula (AAF)	12 months	Infants with moderate-to-severe CMPA fed the study formula with two HMO achieved adequate growth, with some catch-up growth. The formula was safe and well- tolerated.	The gut microbiome characterization demonstrated a significant early enrichment in HMO-utilizing, infant-type bifidobacteria, and later enrichment in *Bacteroides* and butyrate producing taxa in the second half of the first year. Conversely, there was a significant reduction in Proteobacteria. Microbiome changes were associated with a significant rise in fecal SCFA concentrations from enrollment to 12 months of age.
Ramirez-Farias et al. ^153^ 2021	2’-FL	United States	Infants (0–60 days of age) with suspected food protein allergy, persistent feeding intolerance, or presenting conditions where an extensively hydrolyzed formula (eHF)	Hypoallergenic casein-based powdered eHF formula with 0.2 g/L of 2′-FL.	Hypoallergenic casein-based powdered eHF	2 months	The primary outcome was maintenance of weight for age z-score during the study. HF formula with added 2′-FL was well accepted enabling adequate volume to demonstrate a statistically significant improvement of weight for age z-scores. The formula was safe and well tolerated and consumption of the formula over 60 days showed improvement and resolution of persistent symptoms.	Tolerance measures such as mean rank stool consistency, stool color, average volume of formula intake and percent of feedings associated with spit-up/vomit after 1 hr of feeding per day were comparable to other eHF feeding studies that did not contain 2′-FL.
Storm et al. **179** 2019	2‘FL	United States	Healthy, full-term (≥37 weeks gestation; ≥2500 and≤4500 g birth weight), singleton infants, ages 14 ± 5 days, who had been exclusively formula-fed for at least 3 days prior to enrollment were recruited for this trial.	100% whey protein, partially hydrolyzed, contained *B lactis (*0.67 kcal/mL and 2.2 g protein/L).+0.25 g/L 2′FL	100% whey protein, partially hydrolyzed, contained *B lactis (*0.67 kcal/mL and 2.2 g protein/L).	6 weeks	The primary outcome was comparison of Infant Gastrointestinal Symptom Questionnaire, and the scores for the Test and Control group were similar at baseline and during the follow-up.	Anthrpomoteric measurements were similar. Based on data recorded by caregivers in the 2-day diaries, stool frequency and consistency did not differ significantly between groups. Crying and fussing duration and vomiting frequency were similar between groups Average formula consumption volumes did not differ between formula groups. Adverse events were similar, and both formulas are well-tolerated. Reported infections/infestations were lower in 2’-FL group (8% vs. 23%; *p* = .05).
Roman et al. ^156^ 2020	2‘FL + LNnT *L. Reuteri* DSM 17,938	Spain 2018–2019	Healthy, term (37–42 weeks of gestation) infants enrolled at age 7 days to 2 months. *L. Reuteri DSM 17,938*	Exclusively formula-fed group who received a milk-based formula with 2’ FL and LNnT, *L. Reuteri DSM 17,938*	Exclusively breastfed infants, A group mixed fed with both formula and human milk	8 weeks	Formula-fed infants, either exclusively or mixed fed, receiving the HMO-supplemented formula had age-appropriate growth in line with the WHO standards,	The formula was well tolerated, and GI tolerance in the formula- fed infants was comparable to that in breastfed infants.
Kajzer et al. ^157^ 2016	2’-FL and scFOS		Full term, singleton infants (birth weight≥2490 g) enrolled between 0 and 8 days of age.	Experimental Formula 2 contained 2 g/L scFOS and 0.2 g/L 2′FL (*n* = 46).	Experimental Formula 1 did not contain oligosaccharides Breast-fed group.	35 days	The primary outcome was average mean rank stool consistency from Study Day 1 to Visit 3 and there were no differences between the groups.	There were also no differences among groups for predominant stool consistency from Study Day 1 to Visit 3. The average number of stools per day for the HM group was significantly greater than EF1 (*p* < 0.0001) and EF2 (*p* < 0.0001) from Study Day 1 to Visit 3 formula groups. At Visit 3, there were no differences between groups for average volume of study formula intake, number of study formula feedings per day, anthropometric data or percent feedings with spit-up/vomit.
Leung et al. ^166^ 2020	2’-FL	China	Children aged 1–2.5 years (*n* = 461)	2’-FL	Young child formula or one of three new YCFs containing bioactive proteins and/or the HMO 2’-fucosyllactose (2’−FL) and/or milk fat for six months.		There were no significant between-group differences in incidence of upper respiratory tract infection and duration of gastrointestinal tract infections.	

L. reuteri: *Lactobacillus reuteri*, HMO: Human milk oligosaccharide, eHF: extensively hydrolyzed formula, GI: gastrointestinal, BF: breast-fed.

HMO production technologies involve novel processes, which are approved by the regulatory authorities, such as the European Food Safety Agency (EFSA) or the Federal Drug Administration (FDA) in the United States. Both the EFSA in 2015 and the FDA in 2016 approved 2’-FL and LNnT to be added to infant formula, and the first formulas containing HMOs were commercialized in Spain and the USA in 2016. The EFSA indicated that the addition of 2′-FL and LNnT at a ratio of 2:1 to infant formula is safe below 1 -year-old, with a maximum dosage for 2′-FL of 1.2 g/L and for LNnT of 0.6 g/L^20^. In 2019, the FDA stipulated that the maximum dosage of 2′-FL in infant formula is 2.4 g/L, and for LNnT 0.6 g/L^20^. HMOs have obtained the Generally Recognized as Safe (GRAS) status. The number of HMOs that can be synthesized on an industrial scale has steadily increased, and nowadays formulas containing seven HMOs (2’-FL, 3´-FL, LDFT, LNnT, LNT, 3’-SL, 6’−SL) are studied. Some oligosaccharides, identical to those in human milk, can be produced by fermentation or other techniques. To be clear, the oligosaccharides added to infant formula do not originate from human milk, even if they have an identical structure. Therefore, HMOs that do not originate from human milk should preferably be called “human identical milk oligosaccharides” (HiMOs)^4^.

Already in 2005, LNnT was shown to be safe in 228 infants aged 6–24 months during a 16-week follow-up period, with a slight non-significant trend for higher weight and height^148^. Marriage *et a*l^149^ conducted a prospective, randomized, controlled growth and tolerance study, with a formula containing 2′-FL and GOS in healthy full-term infants and showed similar weight, length, and head circumference to breastfed babies from enrollment (0–5 days) to four months^149^. This formula was well-tolerated and comparable for average stool consistency, number of stools per day, and percent of feedings associated with spitting up or vomit with the control group fed GOS supplemented formula. The formula supplemented with 2*′-*FL resulted in a growth similar to that of breast-fed infants^149^. In a multicenter, RCT in Italy and Belgium, Puccio *et al*.^118^ reported the first clinical trial with infant formula supplemented with 2′-FL (1.0 g/L) and LNnT (0.5 g/L) up to the age of 6 months^118^. The 2′-FL and LNnT supplemented formula was well-tolerated and supported age-appropriate growth; infant had softer stools and fewer nighttime wake-ups at two months, while cesarean-born babies had a lower incidence of colic at four months^118^. Infants receiving HMO-containing formula had significantly fewer parent-reported lower respiratory tract infections, antipyretic, and antibiotic use up to the age of 12 months (although the supplementation was limited to the age of 6 months)^118^. Parschat *et al*^160^ conducted a multicenter, randomized, controlled, parallel-group clinical study in Germany, Italy, and Spain to evaluate the safety and tolerability of a five HMO blend (5.75 g/L total, comprising 52% 2′-FL, 13% 3’-FL, 26% LNT, 4% 3′-SL, and 5% 6′-SL) and its effect on growth when applied over a 16-week period^150^. The primary outcome was the mean daily body weight increment over a 4-month period. The observed mean values for daily weight increase of~28.7 g/day were similar to those reported in studies comparing infant formula with 2′-FL plus GOS, 2′-FL plus LNnT, or 2′-FL plus 3′-GL and GOS/FOS^118,149–151^. Lasekan *et a*l^152^ performed a randomized, double-blind, controlled parallel feeding trial with five HMOs (2′-FL, 3-FL, LNT, 3′-SL, and 6′-SL) containing formula in the United States, mostly during the COVID-19 pandemic, while stay-at-home orders were in place^152^. The test formula was again found to be safe and well tolerated and weight gain and length did not differ between the groups. Compared to the control group, infants given test formula had more frequent and softer stools^152^. Vandenplas *et al*.^151^ studied growth, safety, and tolerance in healthy infants consuming a partly fermented infant formula with postbiotics and the HMOs 3′–GL) and 2′-FL, and a specific prebiotic mixture of short-chain GOS (scGOS) and long-chain fructo-oligosaccharides (lcFOS). Equivalence in weight gain (primary endpoint), length, and head circumference gain of up to 17 weeks was also confirmed with the test formula. There were no statistically significant differences between the formula groups for regurgitation, vomiting, watery, or hard stools at any timepoint^151^. Ramirez-Farias and colleagues^153^ examined extensively hydrolyzed formula (eHF) with 2′-FL (0.2 g/L) for growth, tolerance, and compliance in a non-randomized, single-group, multicenter study. Infants (0–60 days old) with suspected food protein allergy, persistent feeding intolerance, or presenting conditions where an eHF was deemed appropriate were enrolled in a 2-month feeding with an experimental formula. This study shows that eHF formula with 2′-FL was well-tolerated and provided a significant improvement of weight for age z-scores^153^. An eHF with two HMOs (2′-FL at 1.0 g/L and LNnT at 0.5 g/L) confirmed a non-inferiority of the test formula for weight gain per day at the 4-month visit, and there were no statistically significant differences between the groups on any of the anthropometric parameters measured during the course of the trial. Gold *et al*.^154^ showed in an open-label, non-randomized, multicenter study of an amino acid-based formula supplemented with two HMOs (2′-FL and LNnT) for 4 months, with the option to continue feeding it for additional 8 months, and showed that the weight-for-age Z score improved from −0.31 at the start of the trial to+0.28 at the end of the study. Additionally, linear and head growth followed the WHO child growth reference and showed a similar, slight upward trend.

#### HMOs in infant formula and gastro-intestinal tolerance

Infant formula supplemented with 2′FL alone, 2′FL combined with LNnT, and a blend of five HMOs (2′-FL, 3-FL, LNT, 3′-SL, 6′-SL) in formula with intact and hydrolyzed protein have all been shown to be well tolerated in clinical trials^118,149,150,153,155–157^. Stool consistency, flatulence, and the frequency of spitting up/vomiting were similar in infants given formula containing with or without HMOs^149,157,158^. In an RCT testing, a mix of 5 HMOs in infant formula, the stools in the HMO supplemented formula group were more soft, frequent, and yellow. They were more similar to the breastfed infants’ stools than the stools of the non-supplemented formula group^150,172^. A formula including 2′-FL and LNnT showed softer stool consistency in another investigation^118^. Stool consistency in infants fed 2′-FL and FOS-containing formula was found to be comparable to that of breast-fed infants^157,158^.

#### The effect of HMOs in infant formula on microbiota composition

There is a significant difference in intestinal microbiota composition between breast-fed infants and formula, without supplementation of biotics, fed infants^136^. Supplementation with HMOs may therefore potentially increase bifidobacteria and bring microbiota composition closer to that of breastfed infants. In RCT, the microbiota composition in a 2’-FL formula (1 g/L) group was only just significantly different at 2 months and just not at 3 months of age, bringing the microbiota composition somehow closer to that of breastfed infants^159^.

The development of the microbiota composition was tested via stool cultures during incubation with 2’-FL of three breast and three formula fed infants^160^. The composition of the microbiome at baseline was dependent on the mode of feeding and on the ability to degrade 2’-FL. When looking at the degradation of 2’-FL, the fecal cultures could be divided into slow and fast degraders regardless of mode of feeding. However, since there were only six infants no conclusions can be drawn^160^. Another multicenter study examined fecal cultures of infants receiving either a formula with a mix of five HMOs at a concentration of 1.5 g/L, a formula with a mix of five HMOs at a concentration of 2.5 g/L, a non-supplemented formula, or breast milk^25^. The microbiota composition of infants receiving formulas supplemented with HMOs was significantly different to those in the non-supplemented group and were closer to the composition of the breastfed infants. The concentration of *B. infantis* was statistically higher in the HMO supplemented than in the non-supplemented group, approaching the composition of breastfed infants. Significantly less *Clostridium difficile* was seen in the HMO supplemented group in comparison to the non-supplemented group suggesting a lesser chance of diarrheal illness. No significant differences were seen between the lower and higher dose HMO supplemented formulas^25^. In a randomized, double-blind, multicenter clinical experiment, after a three-month intervention, infant formula containing 2′-FL and LNnT enhanced the abundance of *Bifidobacterium* and *Streptococcus* and changed the microbiome of cesarean section infants’ group to that observed in vaginal delivery infants^161^. This study suggests that the association between formula with 2′-FL and LNnT and lower parent-reported morbidity and medication use may be linked to gut microbiota community types^161^. A bifidogenic effect in infants receiving formula with two HMOs (2′-FL and LNnT) which was more pronounced in the cesarean-born infants, however, found no effect on *B. infantis*^161,162^. An amino acid-based formula supplemented with 1 g/L 2’FL and 0.5 g/L LNnT confirmed an enrichment in *Bifidobacteria* and reduction of *Proteobacteria*^154^.

Bifidobacteria abundance and metabolic activity could be associated to decreased respiratory tract infections^66,72,163^. Increased gamma-glutamylation and N-acetylation of amino acids, and decreased inflammatory signaling lipids, are the three most notable molecular pathways^66^.

Bosheva *et al*^25^ studied gut maturation effects (microbiota, metabolites, and selected maturation indicators) of an infant formula containing five HMOs (2′-FL, 3-FL, LNT, 3′-SL, 6′-SL). In the first 6 months of life, the HMO supplemented formula shifted the gut microbiome closer to that of breastfed infants with higher bifidobacteria, particularly *B. infantis*, and lower *C. difficile* ^25^. Formula with these 5 HMOs suggest that the HMOs may boost infant intestinal immune development and gut barrier function. HMO-supplemented formula helps restore dysbiosis in cesarean-born infants^25^.

Estorninos and colleagues^164^ evaluated the effects of bovine milk-derived oligosaccharides (primarily composed of GOS with inherent concentrations of sialylated oligosaccharides structurally identical to some in human milk) and reported similar effects on gut microbiota and intestinal immunity in healthy term formula-fed infants^164^.

### Effects of HMOs containing infant formula on infectious disease prevention

Breastfed children are less likely to suffer from respiratory and gastrointestinal infections than formula fed infants^26,98,118,149^. Research with formulas supplemented with HMOs found (as a secondary outcome) a decreased rate of respiratory tract infections and bronchitis, as well as a decreased need for antibiotics and antipyretics^118,161,165^. These effects did persist beyond the six-month intervention period^118,161^. Further analyses of the same data have linked a microbiome community structure highly dominated by Bifidobacterium species at 3 months of age with a decreased need for antibiotics, lending credence to the observation that 2’-FL and LNnT supplementation reduces the risk of respiratory infections and the need for antibiotics^161^. Acetate, one of the compounds produced by the HMO-stimulated metabolic activity of Bifidobacterium, may aid in lowering the risk of respiratory tract infections. Another study found that infants who were fed a formula containing 2′-FL (0.2 g/L) and GOS (2.2 g/L) had a lower incidence of illnesses and infestations as reported by the investigators^149^. Supporting the hypothesis that the HMO-containing formula provides immune system benefits, in the study by Lasekan *et al*.^152^ fewer infants needed to visit a healthcare professional. However, Parschat *et al*.^150^ found no evidence that infant formula containing five HMOs reduced the risk of infection in infants. Leung *et al*.^166^ enrolled 461 infants aged 1–2.5 years in China in an RCT testing three young child formulas containing bioactive proteins and/or 2’-FL and/or milk fat for six months and found no difference in the incidence of upper respiratory or gastrointestinal tract infections between all groups.

In summary: There is theoretical evidence that HMO supplementation in formula fed infants may have beneficial impacts on microbiota composition, immunological function, and other parameters, hence reducing the prevalence of infections. However, clinical data are not unequivocal and no study was powered to evaluate the effect on infections as a primary outcome.

### Effects of HMOs containing infant formula on the immune system

The effects of HMO 2′-FL enriched feeding formulae on immune function biomarkers in term infants were studied^165^. At the age of three months, the groups receiving an HMO-supplemented formula had a higher secretory immunoglobulin A and lower alpha-1-antitrypsin in comparison to the non-supplemented group possibly offering immunological benefits^25^. A randomized, double-blind, controlled growth and tolerance study was conducted with healthy singleton infants who were enrolled by 5 days of age and fed either formula or human milk exclusively from the time of enrollment to the age of 4 months^165^. GOS was given to the control group, whereas GOS plus either 0.2 or 1.0 g/L 2′-FL was given to the study group and compared to the breastfeeding reference group. Concentrations of plasma inflammatory cytokines were 29–83% lower in infants fed formulas with 2’-FL and GOS than did infants fed the control formula including GOS only. Infants whose formula contained 2′-FL showed innate cytokine profiles more similar to those of breastfed infants. Biomarkers of immune functions such as plasma cytokine concentrations, cytokines released by ex vivo stimulation of peripheral blood mononuclear cells (PBMCs), and percentages of major lymphocyte subsets within the PBMCs population were used in this study to demonstrate the impact of 2′-FL-fortified formulas on the developing immune system. 2′-FL reduced the gap in total T lymphocyte proportions between breastfed infants, which is an indicator of improved adaptive immunity. The discrepancies in apoptotic cell percentages between breastfeeding and control groups were also reduced by 2′-FL, especially in CD8+ T cells and CD8+ T cell subset. These results suggest that compared to GOS alone, supplementing infant formula with 2′-FL promotes immunological development and modulation in a way that is comparable to that of breastfed infants^165^.

### Effects of HMOs containing infant formula on allergy

Infants diagnosed with CMPA who are not breastfed are treated with a cow’s milk elimination diet, eHF or amino acid formula^167^. Preclinical studies have indicated that 2′-FL can reduce allergic responses in a food allergy model^120,131^. Laboratory analysis of 2′-FL and LNnT batches showed no evidence of residual milk allergens, despite the fact that HMOs are produced via biofermentation from lactose, which in theory might bring a risk of residual milk allergen contamination^168^.

An eHF with two HMOs (2′-FL at 1.0 g/L and LNnT at 0.5 g/L) showed a similar reduction in the supplemented and non-supplemented eHF, with the Cow’s Milk-Related Symptom Score (CoMiSS^TM^) dropping to the levels seen in presumed healthy infants. Otitis media and upper respiratory tract infections were significantly reduced in the HMO group by 12 months, and lower respiratory tract and gastrointestinal infections were reduced by 30–40%, however without statistical significance^169^.

In an open-label study testing an AAF with two HMOs (2´-FL and LNnT) a significant reduction in symptoms was noted between enrollment and Visit 1, as reported by parents, and between Visit 1 and subsequent visits, as assessed by physicians^154^. Control of skin symptoms was generally excellent.

## Non-human Oligosaccharides

Non-human oligosaccharides were also shown to enhance the development of a bifidobacteria dominated gastrointestinal microbiome^26^. RCTs evaluating GOS/FOS as well as only-GOS enriched formulas have demonstrated a stimulating effect on the growth of *Bifidobacteria* and/or *Lactobacilli* ^26^. GOS, FOS, and GOS/FOS mixtures (the most studied being a 9:1 mixture of scGOS and lcFOS) are the most researched prebiotics components^26,170,171^. Clinical studies have shown that supplementation of infant formula with a mixture of scGOS and lcFOS (9:1) leads to a more favorable gut microbiota composition and activity, closer to that observed in breastfed infants. There was no statistically significant difference between infants fed GOS/FOS enriched formulas and those receiving regular formulas in terms of weight, height, or head circumference^172^. Moreover, scGOS and lcFOS in infant formula has also been associated with a lower number of infections, fever episodes, and antibiotic prescriptions^170,171^. Beneficial effects on *Bifidobacteria* and *Lactobacilli* growth in infants given a scGOS/lcFOS supplemented formula were observed to be sustained even after the formula was discontinued, at least for a few months^172^.

Infant formulae with added prebiotics have been linked to a lower fecal pH and a SCFAs pattern closer to that of breastfed infants, without increased frequency of stool^26^. Non-human oligosaccharides also promote the growth of a bifidobacteria-dominated gut microbiome, selectively stimulate the growth of *Bifidobacteria* and/or *Lactobacilli*^26^. Clinical investigations have demonstrated that adding a mixture of scGOS and lcFOS (9:1) to infant formula results in a more favorable gut microbiota composition and activity, closer to breastfed infants. Beneficial effects on *Bifidobacteria* and *Lactobacilli* growth in infants fed with a scGOS/lcFOS supplemented formula were observed to be sustained even after months of discontinuing the formula^178^. It has been shown that some bifidobacteria only grow in the presence of human milk oligosaccharides^45^. However, it is not known if this has any clinical impact for the infant. There are almost no data comparing the effects of HMO and non-human oligosaccharides in infants. Only in the study by Marriage *et al*.^149^ there was only-GOS group compared to two GOS group with different levels of 2´-FL. As a consequence, there is no evidence to state that HMOs added to infant formula are more effective than non-human oligosaccharides.

## Limitations

Today, there is still a dearth of information on the addition of HMOs to infant formula. No definitive conclusions can be drawn on whether supplemented or non-supplemented formula yields better clinical outcomes because to the limited data from the current research. Due to the differences in design and primary outcomes of the clinical trials, there is inconsistency in the findings. The optimal dosing of HMOs also necessitates fine-tuning. There are substantial variations in the studies in terms of study design, location, lactation sampling, the number of time periods at which development parameters are assessed, the specific HMOs that were analyzed, and the statistical methodologies utilized to predict the correlations^4^. The majority of the included studies have a relatively small sample size to quantify disease outcomes, which reduces their precision and statistical ability to find meaningful relationships. Because of these differences, it is not possible to do a meta-analysis. The benefits of sialylated-HMOs are not well recognized, despite the fact that neutral oligosaccharides like 2′-FL and 3′-FL have been the subject of substantial research into their involvement in infant nutrition, growth, and development in both pre-clinical and clinical settings. There is an immediate need for more investigations on the health advantages of HMOs in human milk with varying structural compositions^136^. There are almost 200 different oligosaccharides in human milk, but today only five are added to infant formula, while studies with seven are going on. An increase in the number of HMOs used could enhance the outcomes. However, the ideal dosage of HMOs in infant formula is still up for debate, as HMO levels fluctuate in breast milk. Therefore, if the formula is administered at a consistent HMO concentration and ratio, formula-fed infants may consume less of specific HMOs in the early stages of the trial, but more HMOs afterward than breastfed infants. The fact that statistical associations do not imply a causal relation further emphasizes the need for randomized, placebo-controlled interventional trials and supplementary mechanistic studies.

In conclusion, HMOs are a major ingredient of human milk, which is the best source of nutrition for infants. HMOs act in tandem with other bioactive components and also act through many pathways that converge to specific activities, as is predicted from many biological processes. HMOs are known to support a healthy gut microbiome, build the gastrointestinal barrier and promote brain growth and cognitive function, among other important physiological roles. A growing body of research also suggests that particular HMOs contribute to the development of immunological competence, both locally and systemically, in part through influencing the metabolism of particular bacteria, such as particular *Bifidobacterium* species. The study of milk microbiota and HMOs relies heavily on the strain-specific characterization of beneficial human microbiota organisms and their consumption of specified HMOs. Human milk research is a promising field since more benefits and correlations between components will be uncovered as time goes on. Regarding formula feeding, more clinical trials in children are needed comparing the multiple effects of non-human to human oligosaccharides supplementation.
